# Sequential Differentiation of Embryonic Stem Cells into Neural Epithelial-Like Stem Cells and Oligodendrocyte Progenitor Cells

**DOI:** 10.1371/journal.pone.0155227

**Published:** 2016-05-18

**Authors:** Jing Bian, Jiao Zheng, Shen Li, Lan Luo, Fei Ding

**Affiliations:** 1 Jiangsu Key Laboratory of Neuroregeneration, Collaborative Innovation Center of Neuroregeneration, Nantong University, Nantong, Jiangsu, China; 2 Xijing Hospital, The fourth Military Medical University, Xi’an, Shanxi, China; 3 Department of Gerontology, Affiliated Hospital of Nantong University, Nantong, Jiangsu, China; Instituto Cajal-CSIC, SPAIN

## Abstract

**Background:**

Recent advances in stem cell technology afford an unlimited source of neural progenitors and glial cells for cell based therapy in central nervous system (CNS) disorders. However, current differentiation strategies still need to be improved due to time-consuming processes, poorly defined culture conditions, and low yield of target cell populations.

**Methodology/Principle Findings:**

This study aimed to provide a precise sequential differentiation to capture two transient stages: neural epithelia-like stem cells (NESCs) and oligodendrocytes progenitor cells (OPCs) derived from mouse embryonic stem cells (ESCs). CHIR99021, a glycogen synthase kinase 3 (GSK-3) inhibitor, in combination with dual SMAD inhibitors, could induce ESCs to rapidly differentiate into neural rosette-like colonies, which facilitated robust generation of NESCs that had a high self-renewal capability and stable neuronal and glial differentiation potentials. Furthermore, SHH combined with FGF-2 and PDGF-AA could induce NESCs to differentiate into highly expandable OPCs. These OPCs not only robustly differentiated into oligodendrocytes, but also displayed an increased migratory activity *in vitro*.

**Conclusions/Significance:**

We developed a precise and reliable strategy for sequential differentiation to capture NESCs and OPCs derived from ESCs, thus providing unlimited cell source for cell transplantation and drug screening towards CNS repair.

## Introduction

The adult central nervous system (CNS), including the brain and spinal cord, has a limited self-repair capability after injury [1,2]. The endogenous neural stem cells (NSCs) in the adult brain can contribute to nerve regeneration after injury due to their neuronal and glial differentiation potential, but the cell number is not large enough to achieve the therapeutic goal after injury [3–6]. Besides stimulation of endogenous NSCs, therefore, transplantation of exogenous NSCs becomes another important approach for cell-based therapy of CNS pathology because endogenous NSCs can also differentiate into functional neurons and glial cells in vitro and in vivo to help rebuild the damaged neural tissue in response to nerve injury [3].

Recent development in stem cell technology may provide an unlimited cell source for stem cell-based therapy in CNS disorders [5,7,8]. Current neural differentiation strategies, however, are hindered by poorly defined culture conditions [9], protracted differentiation to obtain highly homogenous stem cell populations, and even relatively low yield of target cell populations [10–13]. For example, NSCs, obtained either from fetal tissues or embryonic stem cells [1], can be used to treat CNS disorders [2,4,14,15], but these cells often lack high purity and homogenous properties depending on the protocols of generating NSCs [2].

The ideal neural stem/progenitor cells derived from pluripotent stem cells can effectively expand and undergo neuronal and glial differentiation in vitro [11] [16]. The goal of our study was to establish a precise and sequential differentiation strategy to control the generation of homogeneous NSCs and neural glial progenitors derived from embryonic stem cells under the regulation by distinct signaling pathways. In this study, we wanted to recapitulate the signals involved in epidermal formation and subsequent neural patterning [5,12,17] and induce the differentiation of mouse embryonic stem cells (ESCs) towards the neural fate by using the corresponding growth factors and cytokines. The successful development of this strategy could provide a new and reliable model system for studying neural development, and produce unlimited neural stem/progenitors for drug screening and cell transplantation in treating CNS disorders.

There are extensive studies focusing on neural differentiation from human pluripotent stem cells, but the long-term process of differentiation greatly limits its applications. For instance, it takes almost 6 months to generate oligodendrocyte progenitor cells (OPCs) [8], or 3–4 months to generate long-term self-renewing neuroepithelial-like stem cells through embryoid body differentiation [12]. Since the process to develop mouse embryo is much shorter than that to develop human embryo, we speculated that it took much less time to identify the key differentiation factors that control neural differentiation from mouse ESCs than from human MSCs. It is worth to mention that mouse ESCs share some features and developmental clues with human ESCs or human induced pluripotent stem cells (iPSCs) [5], but the pluripotency of mouse ESCs is categorized into a primitive, naïve LIF-dependent state that is distinct from a primed bFGF-dependent state represented by human ESCs or mouse epiblast-stem cells (EpiSCs) [18,19]. And there are very few protocols describing a direct neural differentiation of mouse ESCs into homogenous NSCs and then glial cells [16]. In this study, we anticipated that a precise control of defined signals would allow a robust and specified generation of neural lineage cells from pluripotent stem cells in a primitive, naïve LIF-dependent state.

## Materials and Methods

All experimental procedures involving animals were conducted as per institutional animal care guidelines of Nantong University and were approved ethically by the Jiangsu administration committee for experimental animals, China.

### Harvesting and culture of mouse ESCs and mouse NSCs

The mouse ESCs (M1, passages 21 to 40) were gifted by Dr. Lei Xiao at Zhejiang University (Hangzhou, China). Details can be found in S1 File. The mouse NSCs were isolated from pregnant ICR mice and cultured (see S1 File).

### Differentiation of ESCs into neuroepithelial-like stem cells (NESCs)

The mouse ESCs were dissociated as single cells using accutase (Gibco, Grand Island, NY), and cultured on 5% Matrigel (BD Biosciences, Two Oak Park, MA) coated plates with a feeder-free culture medium before induction. On day 1, media were replaced by NE media 1 consisting of DMEM /F12 supplemented with 1×B27, 1×N2, 1×NEAA, 0.1 mM β-Me (all 4 products from Gibco), 100 ng ml^-1^ Noggin (R&D Systems, Minneapolis, MN), 20 μM SB431542 (Sigma, St. Louis, MO), 2 μM dorsomorphin (Sigma), and 3 μM CHIR99021 (Sigma). On Day 4, neural rosettes-like colonies were released using 1.5 mg ml^−1^ collagenase IV (Gibco), and fed with NE media 2 consisting of DMEM /F12 supplemented with 1×N2, 20 ng ml^-1^ FGF2, 1.6 g L^-1^ glucose (Sigma) from non-coated plates for additional 4 days. On day 8, neurospheres were collected, treated with accutase, and transferred to matrigel-coated plates for culture in NE media 3 consisting of DMEM /F12, supplemented with 1×N2, 1 μl ml^-1^ B27 (1:1000), 20 μg /ml Insulin, 1.6 g L^-1^ glucose, 20 ng ml^-1^ EGF, 20 ng /ml FGF2 (all from R&D Systems). At 1 day after replating, almost all dissociated cells from neurospheres were attached to the plate as NESCs, which were quickly cultured in the NE media 3 to undergo cell expansion and differentiation toward neurons (S3 Fig) and OPCs.

### Differentiation of NESCs into OPCs

On day 9, ESCs-derived NESCs were dissociated to a single cell suspension using accutase. The cells were counted and plated at 4×10^4^ cells cm^−2^ on plates coated with 0.1 mg ml^−1^ poly (L-ornithine) (Sigma) followed by 10 μg ml^−1^ laminin (Sigma) coating, or coated only with matrigel. Cells were grown in OPC differentiation media (DMEM /F12, supplemented with 1× B27, 1× N2, 0.4 μM SAG (a substitute for SHH [20,21]) (Enzo Life Sciences, Farmingdale, NY) or 200ng ml^-1^ SHH (R&D system), 20 ng ml^-1^ FGF2, 20 ng /ml PDGF-AA (PeproTech, Rocky Hill, NJ) for 5 days. The generated OPCs were grown on pro-laminin or matrigel coated plate. OPCs also expanded in OPC media and were ready to differentiate toward oligodendrocytes (OLs) and astrocytes. All media were replaced every two days. NESCs and OPCs were readily frozen or thawed and were cryopreserved in media with 90% FBS (Gibco) and 10% DMSO (Sigma).

### Differentiation of OPCs into OLs and astrocytes

For differentiation of OPCs into OLs, cells were seeded at 2 × 10^4^ cells cm^−2^ on Matri-gel coated plates and induced differentiation under OL media (DMEM /F12, supplemented with 1×B27, 1×N2 30 ng ml^-1^ T3 (Sigma), 0.4 μM SAG, 100 ng /ml Noggin, 10 μM dibutyryl cyclic-AMP sodium salt (Sigma), 100 ng /ml IGF-1 (PROSPEC, East Brunswick, NJ), and 10 ng/ml NT3 (R&D Systems) for 3 days. Astrocytes were induced with Astro media (DMEM /F12, supplemented with 1× B27, 1× N2, and 20 ng ml^-1^ BMP4) for 7 days.

### Differentiation of NESCs into neurons

For differentiation of NESC into neurons, cells were plated at 4 × 10^4^ cells cm^−2^ and induced neural differentiation under neuron-differentiation medium in DMEM F12 base medium supplemented with B27, N2, and 300 ng ml^-1^ cAMP as described previously [11].

### Growth curve

**Fig 1:**
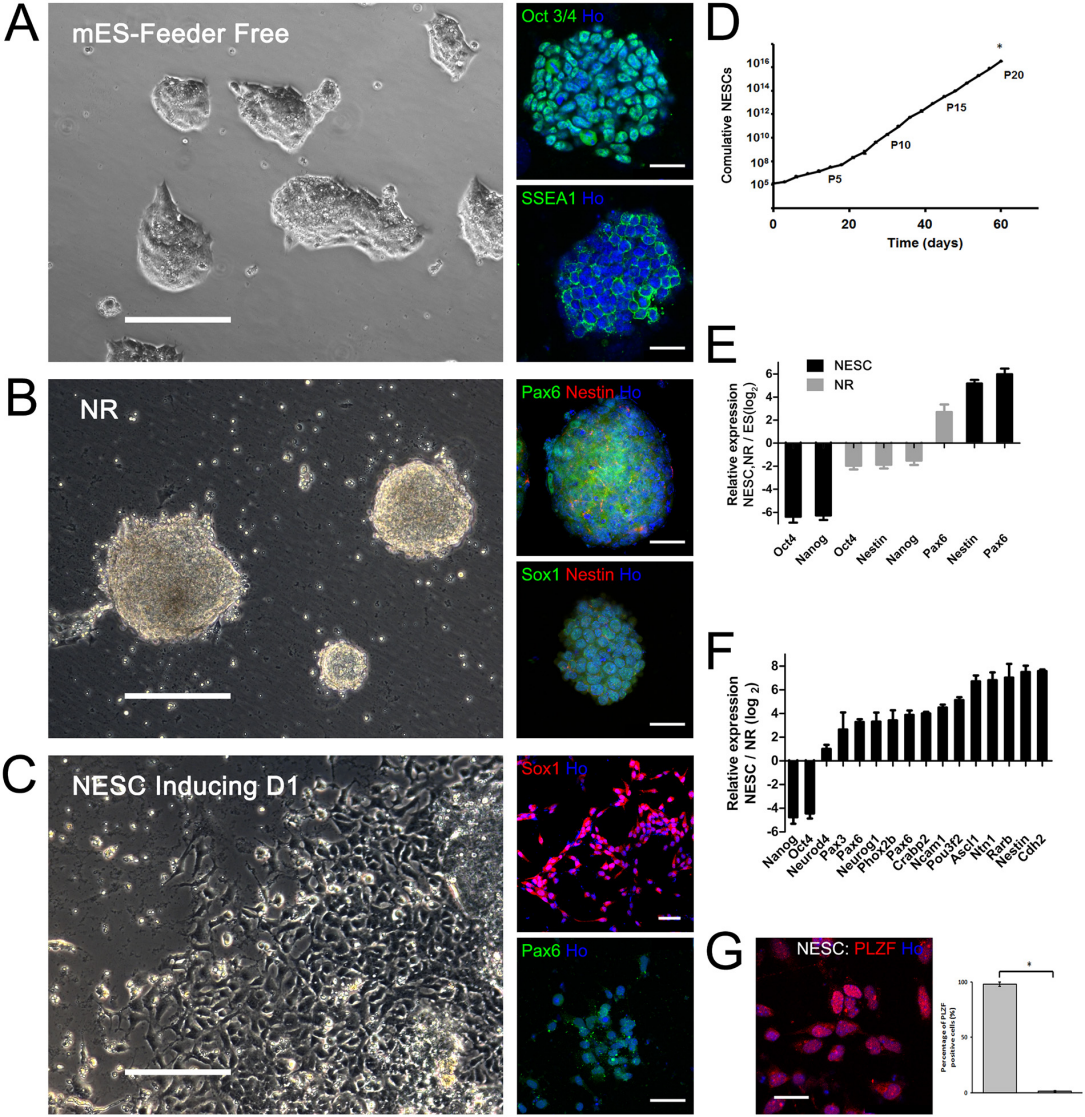
Rapid and robust differentiation of ESCs into NESCs. (A) Phase-contrast and fluorescence images of mouse ESCs, which express Oct3/4 and SSEA1. (B) Under defined differentiation conditions by adding SB431542, Dorsomorphin, Noggin, and CHIR99021, ESCs made transitions into neural rosette-like colonies expressing Pax6 and Sox1. (C) ESCs-derived-NESCs differentiated into neuroepithelial-like stem cells (NESCs) expressing Pax6 and Sox1. (D) Cumulative curve showing ESCs-derived NESCs could be expanded over 20 passages (Passage denoted P1-P20). *p<0.05, passage 20 (P20) versus all other passages. (E, F) qPCR of representative markers during neural differentiation from ESCs to patterned neural rosettes-like colonies (NR) and NESCs showing a rapid downregulation in pluripotency genes such as Oct4 and Nanog and upregulation in genes specific to NR and NESCs such as Pax6 and Nestin. (G) Representative confocal image showing NESCs were highly pure as the percentage of PLZF-positive cells in total cell population was 97% ± 1%, the values significantly higher than those for negative control (cell population stained only with secondary antibody). Nuclei counterstained with Ho (Hoechst 33342 blue). Data were expressed as means ± SEM from 4 chosen fields per slide. Scale bar, 200 μm in phase contrast images and 50 μm in fluorescence images of A, B, C; and scale bar, 25μm in F. *p<0.05.

**Fig 2:**
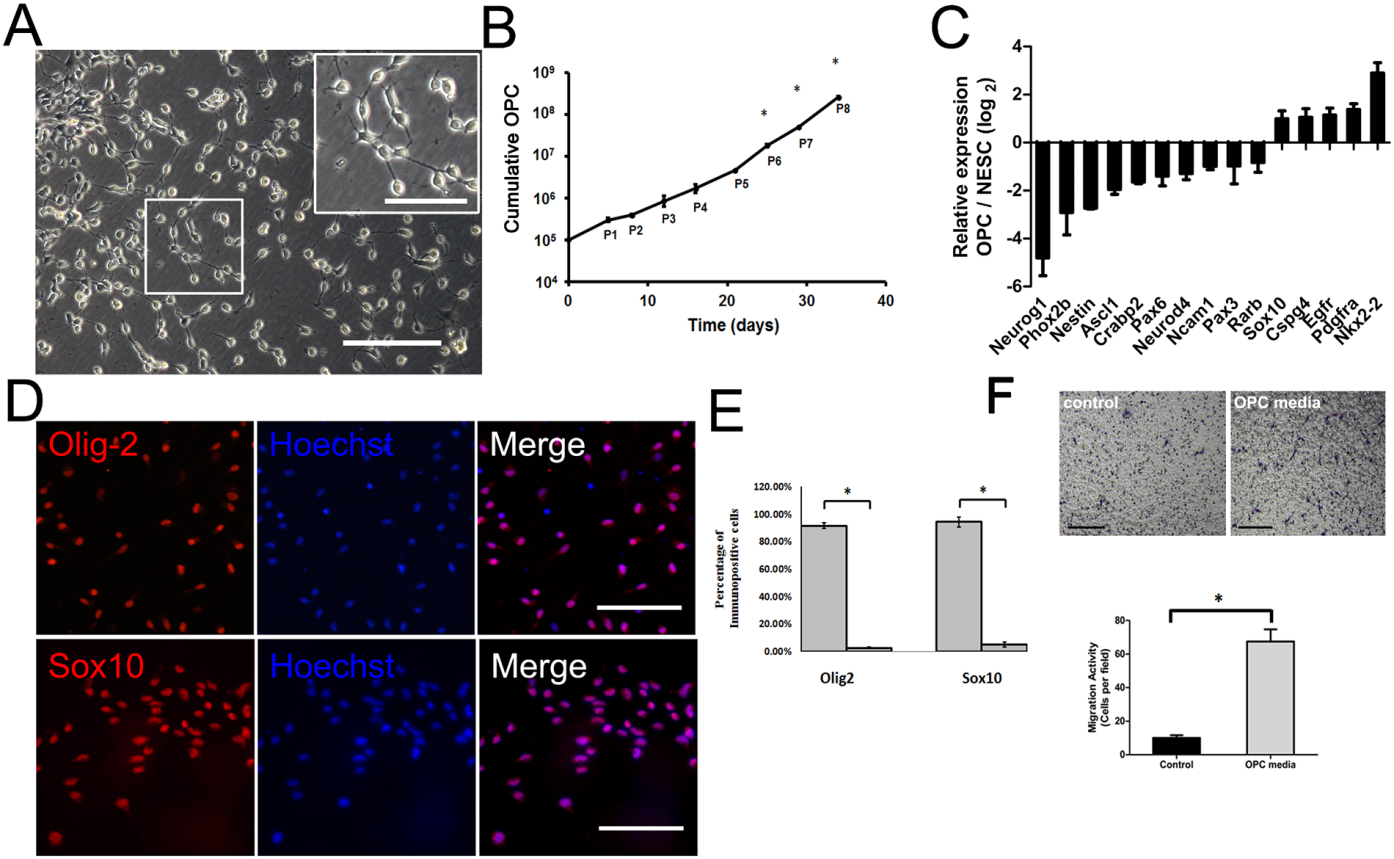
Differentiation of ESCs-derived NESCs into expandable OPCs. (A) Phase contrast image of mESC-derived NESCs differentiated into OPCs with bipolar morphology under defined medium containing FGF2, PDGF-AA and SAG on matri-gel coated plate. Also shown (inset) is a magnification of the boxed area in (A). (B) Cumulative growth curve of NESCs-derived OPCs divide over eight passages (P1-P8). *p<0.05, P6, P7, or P8 versus P1-P5 respectively. (C) qPCR analysis showed that during the transition of NESCs into OPCs, the genes specific to NESCs such as Nestin and Pax6 were down-regulated, while the genes specific to OPCs such as Nkx2.2, Sox10, and PDGFRα were up-regulated. (D) Immunostaining of OPCs showed they were nearly homogenous expressing transcription factors Olig2 and Sox10, (E) The percentages of Olig2- and Sox10-positive cells in total cell population were 91 ± 2% and 94 ± 3%, respectively, the values significantly higher than those for negative control (cell population stained only with secondary antibody). *p<0.05. (F) NESCs displayed a 5–6 fold increase in migratory activity in response to FGF2, PDGF and SHH treatment comparing to the basal medium. Data were expressed as means ± SEM and derived from 2–4 independent experiments, *p<0.05. Scale bar, 200 μm in (A) and 100 μm in (D) or in the inset of (A).

To obtain a long-term proliferation curve, the cumulative cell numbers of NESCs (Fig 1D) and OPCs (Fig 2B) were calculated based on the formula C^n^ = C^n−1^×T^n^/S^n^, where C, cumulative cell number; n, passage number; T, total cell number at current passage; S, seeded cell numbers at current passage. To evaluate the cell growth rate, cells were seeded at 4 × 10^4^ cells cm^−2^, and passaged at 80–90% confluence. Total cell number at each passage was counted with a hemocytometer. Rates of growth were determined at each passage and extended to the entire population of cells to yield a cumulative count.

### RNA isolation, RT-PCR and quantitative real time RT-PCR (qPCR)

RT-PCR and qPCR conditions are described in S1 File. RT-PCR and qPCR primer sequences are listed in S1 and S2 Tables respectively.

### Immunocytochemical staining

Immunocytochemical staining is described in S1 File, and the antibodies used are listed in S3 Table.

### Flow cytometry analysis

The percentage of NESC at passage 1(P1) and passage 10 (P10) after differentiation from ESCs was analyzed under flow cytometry with standard procedures as described in S1 File.

### Migration assay

Migratory activity of ESCs-derived NESCs was assessed with a 24-well transwell chamber system as described previously [22]. The detailed procedure can be found in S1 File

### Statistics analysis

Data were presented as means ± SEM. Statistically significant differences between two groups was assessed by Student's t-test, while those between multiple groups were evaluated by one-way ANOVA test plus Scheffer post hoc tests with the help of SigmaStat 3.5 software (Sigma). *P* < 0.05 was considered significant.

## Results

### 1. Sequential differentiation of ESCs into highly proliferative NESCs

We referred to several published protocols [5,10,23,24], which were used for differentiation of mEpiSCs into neuroepithelium by dual inhibition of SMAD signaling with SB431542 and dorsormorphin (or LDN193189) in combination with noggin (a BMP antagonist). These three inducers, however, failed to induce differentiation of ESCs into NSCs due to the poor attachment and proliferation capability of generated cells (S1 Fig). In order to improve the survival rate and proliferation capability of ESCs-derived NSCs, we added an inhibitor of glycogen synthase kinase 3 (GSK3) to the differentiation medium containing the above 3 inducers, because inhibition of GSK3 signaling might enhance proliferation of neuronal progenitors by modulating Wnt/β-catenin signaling [24–27]. It is well shown that CHIR99021, as a highly selective small molecule inhibitor of GSK3 [28], facilitated the survival of neural precursors converted from hESCs [29]. Therefore we induced ESCs to undergo neural differentiation in the presence of CHIR99021 and other 3 inducers (SB431542, dorsormorphin, and noggin). Importantly, we selected 3 μM as an optimized dose of CHIR99021, and found that a combination of 4 inducers could efficiently induce ESCs to differentiate into highly expandable NESCs through a 3-stage differentiation protocol (Fig 1).

The first stage was differentiation from ESCs to neural rosette-like colonies, the second stage was differentiation from neural rosette-like colonies to neurospheres, and the third stage was differentiation from neurospheres to NESCs which were highly proliferative (S2 Fig). CHIR99021 combined with SMAD inhibitors were added at the first stage to induce a rapid generation of neural rosette-like colonies. Glucose and bFGF were added at the second stage to maintain the growth of neurospheres. EGF and bFGF were added at the third stage to promote cell proliferation of NESCs (S2 Fig).

During these stages of differentiation, ESCs were under morphological changes from embryonic stem cells (Fig 1A) into neural rosette-like colonies (Fig 1B), which latere xpanded into NESCs (Fig 1C). Immunocytochemistry showed that during the 3 stages ofdifferentiation, pluripotency genes, such as Oct3/4 and Nanog, were expressed in ESCs, while neuroepithelial markers, such as Pax6 and Sox1, were expressed in both neural rosette-like colonies (Fig 1B) and NESCs (Fig 1C). The expansion capability of ESCs-derived NESCs was tested by a growth curve assay, which indicated that no change in the growth rate was observed until up to 20 passages, as evidenced by the curve slope (Fig 1D). qPCR data revealed that in neural rosette-like colonies and NESCs, the expression of pluripotency genes, such as Oct4 and Nanog, was dramatically down-regulated, while the expression of neuroepithelial markers, such as Pax6 and Nestin, was up-regulated, compared to that in ESCs (Fig 1E and 1F), providing further evidence for the above immunocytochemistry data. The percentage of PLZF (a marker of NESCs)-positive cells in total cell population was high up to 97 ± 1% (Fig 1G), suggesting that our developed differentiation strategy was very robust and efficient. And the difference between the percentage of PLZF positive and negative control (cell population stained only with secondary antibody) was significant (*p* < 0.05). (Fig 1G)

**Fig 3:**
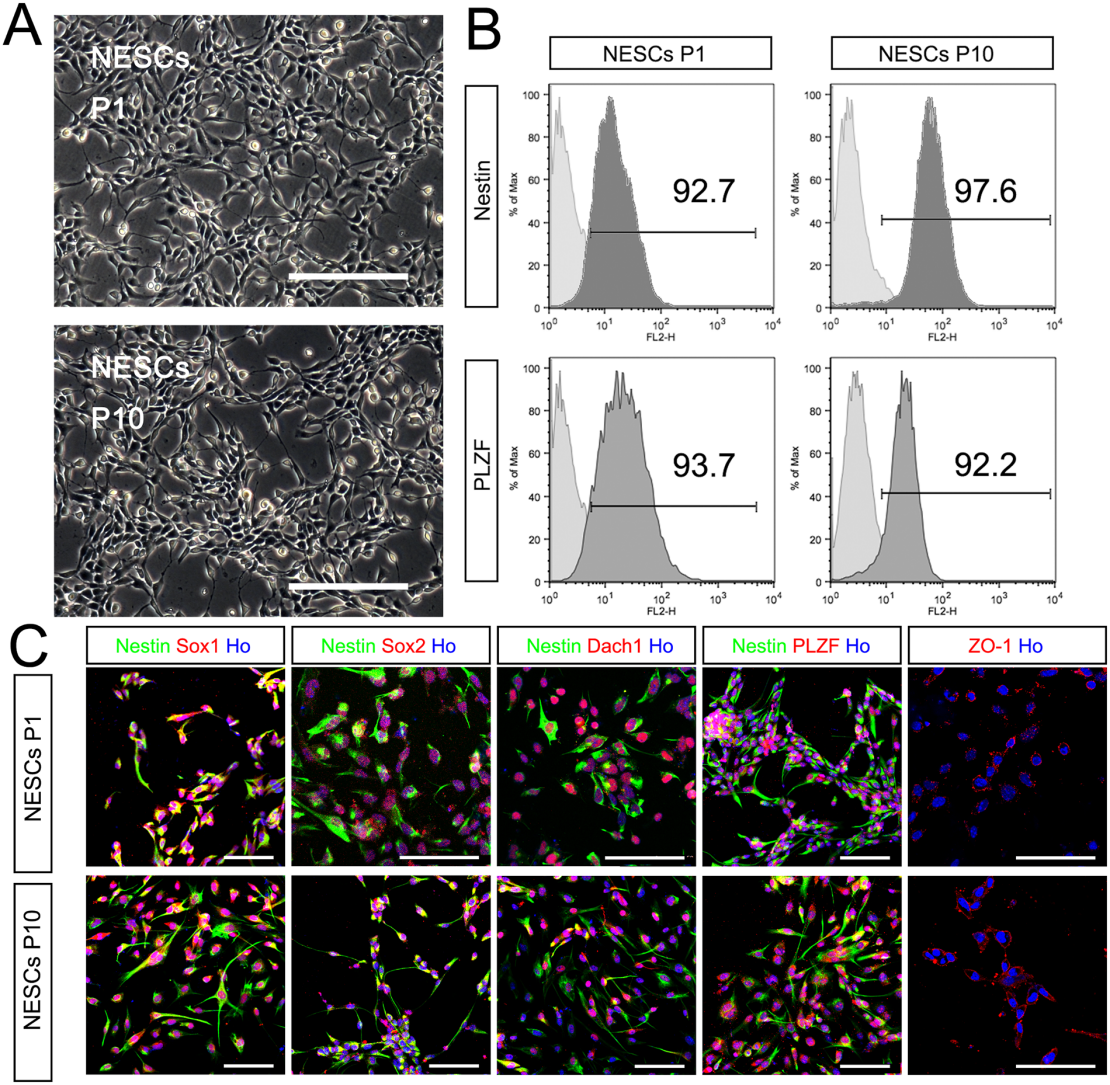
Comparison of NESCs at different passages. (A) Phase contrast of NESCs at P1 and P10. (B) Flow cytometry analysis revealed that percentage of NESC population positive for Nestin and PLZF are over 90% at both p1 and p10. (C) Immunocytochemistry of NESC showed that NESCs at P10 still expressed neuroepithelial stem cell markers, such as Sox1, Sox2, Dach1, PLZF and ZO-1 as in P1. Scale bar, 200 μm in A, and 50 μm in C.

In order to evaluate the stability and homogeneity of ESCs-derived NESCs after continuous passage in culture, we compared the expressions of characteristic markers of NESCs, including neural precursor cell markers (Nestin and Sox2) and neural progenitor transcription factors Sox1 and Pax6 [12], between NESCs at passage 1 (P1) and passage 10 (P10) (Fig 3A). Flow cytometry revealed that NESCs at passage 10 still expressed Nestin (a NSC marker) and PLZF (a neural rosette marker) (Fig 3B). Immunocytochemistry showed that NESCs at passage 10 still expressed Sox9, Sox1, and Sox2 (NSC markers) [13] and also expressed DACH1, PLZF and ZO-1 (neural rosette markers) (Fig 3C). Of note, both NESCs at p1 and p10 expressed ZO-1, which, as a representative neural rosette tight junction marker, is expressed apically at the lumen surface of neuroepithelium to reflect polarization [11,12] (Fig 3). Taken together, all the data confirmed that ESCs-derived NESCs maintained the properties of neural epithelial-like stem cells although they were highly expanded in the presence of bFGF and EGF.

**Fig 4:**
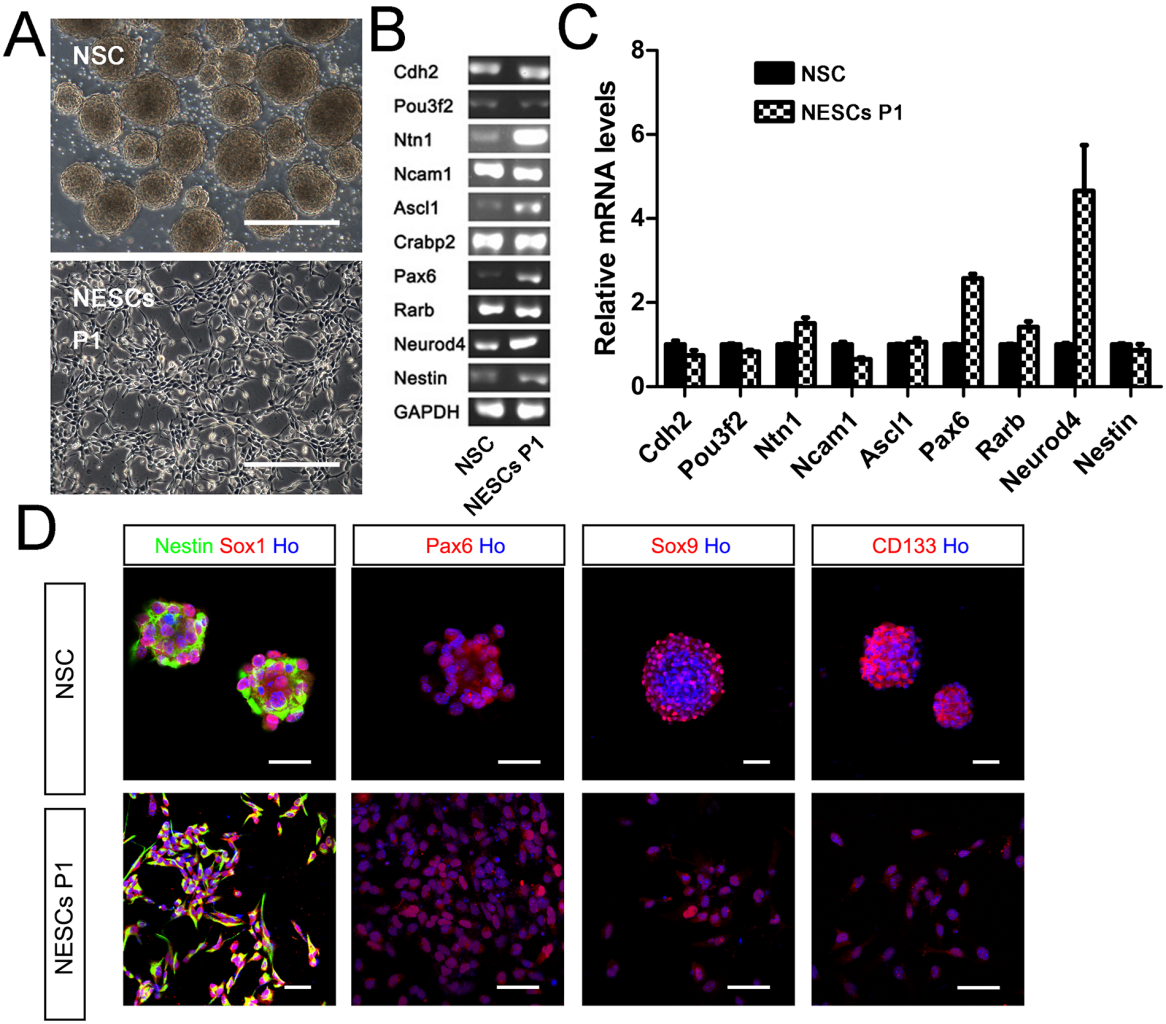
Comparison of NSCs and NESCs. (A) Phase contrast of NSCs isolated from mouse embryo cortex and NESCs derived from ESCs, which were both at p1. (B) RT-PCR analysis of NSCs and NESCs at P1 with representative NSC markers (C) According to the expression of representative genes in NESCs and NSCs, as determined by RT-PCR, NESCs shared similar markers as NSCs such as Pax6, Neurod4, Ncam1, Nestin, and Rarb. (D) Immunocytochemistry of NSCs and NESCs at P1 revealed that they shared similar NSC markers such as Sox1, Pax6, Sox9 and CD133. Scale bar, 200 μm in A, and 50 μm in D.

Next, we investigated whether ESCs-derived NESCs shared the same features as NSCs and whether they can serve as a potential therapeutic candidate for the treatment of CNS disorders. In this study, NSCs were isolated from mouse embryonic cerebral cortex at E13.5 d (Fig 4A). Therefore, we compared ESCs-derived NESCs with NSCs either by RT-PCR (Fig 4B and 4C) or by immunocytochemistry with representative NSC markers (Fig 4D). Immunocytochemistry showed that both NESCs and NSCs expressed the same neural stem cells markers, such as Sox1, Sox9, and CD133 (Fig 4D). It was shown that both cells expressed the same neuroectoderm genes, such as Nestin, Ncam1, Rarb, and Neurod4, and also expressed the neural rosette gene, such as Pax6 [12] (Fig 4B and 4C).

### 2. Neural differentiation of NESCs into OPCs

Since ESCs-derived NESCs shared the same characteristics as NSCs, it is possible to develop applicable protocols for differentiation of NESCs into OPCs and then to provide a renewable source of OPCs.

The differentiation of ESCs-derived NESCs into OPCs was performed according to the previously published protocol for differentiation of neuroectoderm into OPCs [22] with a little modification. In the defined differentiation medium containing PDGF-AA, FGF-2 and SHH, SHH was replaced by smoothened agonist (SAG) [20,21] mainly for cost reduction purpose. After 4–5 day differentiation in the above multi-component medium, ESCs-derived NESCs were quickly transformed into OPCs with bipolar morphology (Fig 2A, S3 Fig). The generated OPCs could expand into homogenous population through at least 8 passages, thus forming a larger number (over 10^8^) of pure OPCs than ever before (Fig 2B). These ESCs-derived OPCs were uniformly immunopositive for Olig2 and Sox10, two markers of OPCs (Fig 2D). The percentages of Olig2- and Sox10-positive cells in total cell population were 91 ± 2% and 94 ± 3%, respectively, the values significantly higher than those for negative control (cell population stained only with secondary antibody) (Fig 2E). In addition, qPCR analysis showed that the expression of NESC marker genes (Pax6 and Nestin) was down-regulated while the expression of OPC marker genes (Sox10, Nkx2.2 and PDGFR-α) was up-regulated in the generated OPCs as compared to that in NESCs (Fig 2C).

After ESCs-derived NESCs were cultured in the plain medium at the presence or absence of FGF2, PDGF and SAG, the cell migration ability was determined. The treatment of cells with the above-mentioned 3 inducers led to a significant (about 5–6 fold) increase in the cell migratory activity as compared to treatment without 3 inducers (Fig 2F). (p<0.05)

### 3. Differentiation of NESCs-derived OPCs into OLs

**Fig 5:**
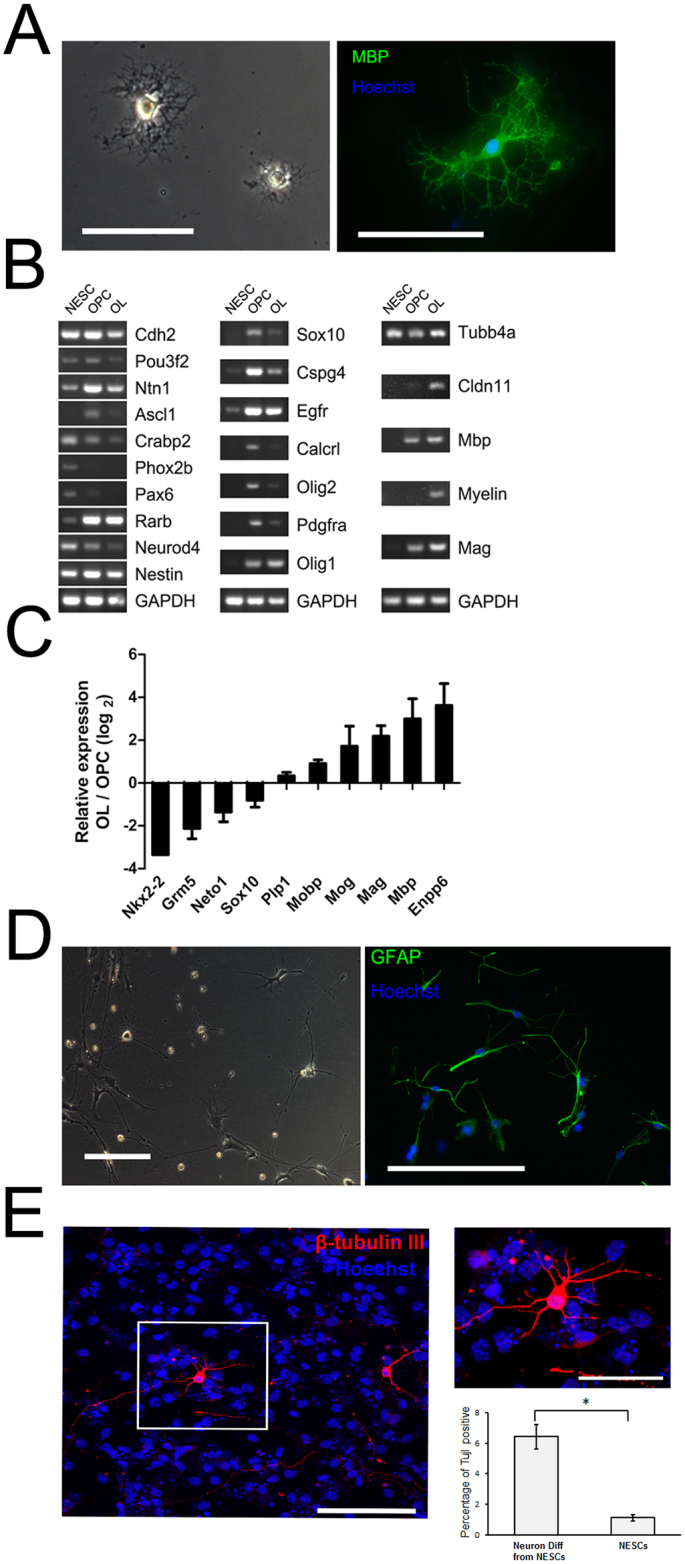
Efficient differentiation of NESCs-derived OPCs into OLs. (A) phase contrast image (left) and fluorescence images (right) by marker MBP for OLs. The percentage of MBP positive population among the differentiated OLs was significantly higher than negative control (cell population stained only with secondary antibody) (B) RT-PCR analysis compared NESCs, OPCs, and OLs with representative markers for each population. (C) qPCR profiling, performed during the transition of NESCs-derived OPCs to OLs, showing a rapid down regulation of OPCs genes and up regulation of genes specific to OLs. (D) Phase contrast (left) and immunofluorescence image (right) of astrocytes marked with GFAP differentiated from OPCs, and quantitative analysis revealed that there was significant different between the percentage of GFAP positive astrocytes and negative control marked only with 2° antibody. (p<0.05) (E) Immunofluorescence image of neurons derived from NESCs with a marker of β-tubulin III. Also shown (at the right half) is a magnification of the boxed area in the left half. Scale bar, 100 μm in (A), 200 μm in (D), 100 μm in the left half of (E), and 50 μm in the right half of (E). *p<0.05.

In order to determine the differentiation capacity of NESCs-derived OPCs into OLs, we treated the cells with a combination of thyroid hormone (T3), NT3, cAMP, IGF-1, and Noggin without using FGF2 and PDGF-AA, which had been used for the differentiation of mEpiSCs-derived OPCs into OLs [5]. During the 4 days’ differentiation, we found that mEpiSC-derived OPCs stopped proliferation accompanied by majority of cell death (around 90%) and among the survivals 40% was transformed into cell populations positive for myelin basic protein (MBP), a representative marker of bona fide OLs **(**Fig 5A). And there was a significant difference between MBP positive cell population and those for negative control (cell population stained only with secondary antibody) (p<0.05) (Fig 5A). RT-PCR analysis showed that NESCs, OPCs, and OLs expressed their respective representative markers (Fig 5B). qPCR analysis revealed that in the generated OLs, the expression of OPC-specific genes (Nkx2.2 and Sox10) was dramatically down-regulated, while the expression of OLs-specific genes, including myelin-associated glycoprotein (Mag), myelin oligodendrocyte glycoprotein (Mog), and myelin basic protein (Mbp) [30], was up-regulated as compared to that in OPCs (Fig 5C).

To assess differentiation capability of OPCs into astrocytes, OPCs were cultured in BMP4–containing neural base medium as described previously [5], and the majority (nearly 60–70%) of NESCs-derived OPCs differentiated into astrocytes that were characterized by GFAP, a specific marker of astrocytes (Fig 5D). The percentages of GFAP-positive cells in total cell population were 63 ± 5%, the values significantly higher than those for negative control (cell population stained only with secondary antibody). *p<0.05.

**Fig 6:**
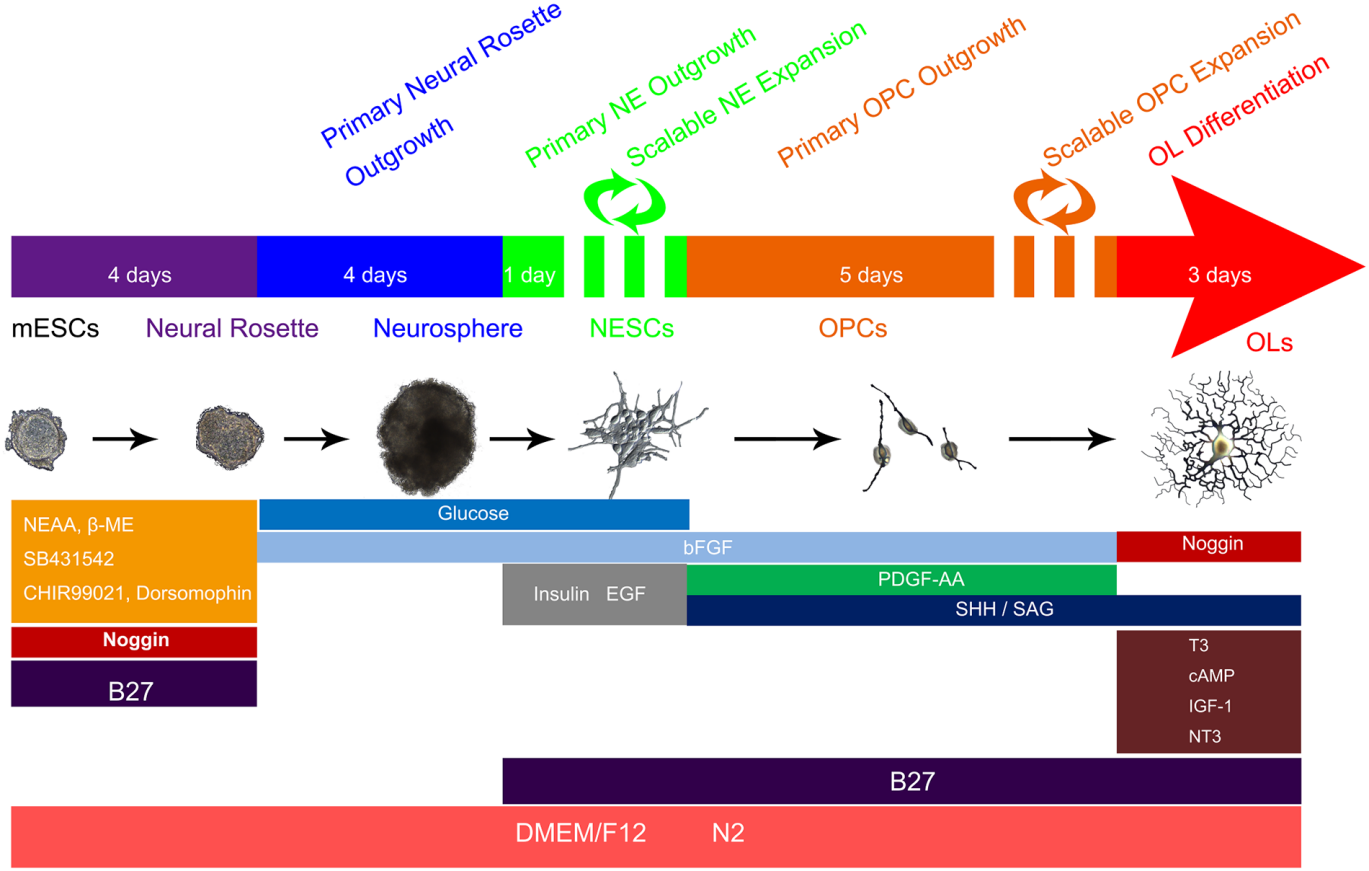
Schematic diagram showing step-by-step differentiation of ESCs into OLs under influences of a series of developmental signals.

Collectively, a well-defined differentiation strategy led to the generation of two transient stages, NESCs and OPCs, during the induced differentiation of ESCs towards OLs (Fig 6**)**.

### 4. Differentiation of NESCs into neurons

We also investigated the possibility of NESCs to differentiate into neurons. NESCs were under differentiation in basal neuron differentiation medium as described previously [11]. After culturing for 10 days, NESCs differentiated into neurons, which were immunopositive for β-tubulin, a neuron marker (Fig 5E). And there was a significant higher percentage of β-tubulin positive neurons than undifferentiated NESCs. (p<0.05) (Fig 5E).

## Discussion

In this study, we developed a robust and direct strategy for sequential neural differentiation of ESCs to give rise to NESCs and OPCs with relatively high proliferation capability. The ESCs-derived NESCs shared the same characteristics as NSCs and had neuronal and glial differentiation potential, suggesting a high-degree of similarity between NESCs and NSCs. Both ESCs-derived NESCs and NESCs-derived OPCs had over 90% purity as evidenced by immuocytochemistry with respective typical markers. Furthermore, our mESC-derived NESCs maintained the expression of typical markers of neural rosettes (the most primitive form of NSCs), and they could differentiate into β-tubulinIII-positive neurons.

Under our well-defined conditions, ESCs could also differentiate into a pure population of expandable OPCs within 14 days without forming embryoid bodies. The NESC-derived OPCs were capable of differentiating into OLs in a high efficient way. The currently available protocols for inducing human pluripotent stem cells into OPCs and OLs in vitro are often inefficient and time-consuming (about 120–150 days) [8], and even using a recently published improved protocol, OPCs are still harvested through 75 days differentiation of hiPSCs [31]. In contrast, our differentiation protocol was more quick and efficient for generating highly proliferative NESCs and expandable OPCs.

In this study, CHIR99021 (a GSK-3 inhibitor), in combination with dual SMAD inhibitors, played an important role in neural conversion of ESCs. CHIR99021 increases the survival of human ES cells-converted neural precursors [29], and induces a rapid and efficient specification of regionally defined human neural progenitors with hindbrain markers, as described previously [32]. In this study, ESCs-derived NESCs, just like neuroepithelial cells that appear transiently in the caudal neural tube at E10.5 during rodent embryo development [33], were hindbrain specification amenable to regional patterning [11]. By using this clue we successfully captured highly-proliferative NESCs when 3 μM of CHIR99021 was used in the first stage of differentiation. CHIR99021 inhibition of GSK-3, combined with SMAD dual inhibition, could facilitate the generation of neural progenitor cells (NPCs). This finding was consistent with previous results about human blood fate to NPCs by reprogramming coupled with Oct4 induction [27]. It should be mentioned that in our protocol, CHIR99021 was used only at the first stage of differentiation. This made our protocol better than the previous protocol [27].

As is known, NESCs are difficult to obtain because of its transient nature [10, 11] during neural differentiation from pluripotent stem cells to OLs. Based on our proposed step-by-step differentiation, however, the generated NESCs could expand in vitro for more than 20 passages, which, in turn, could differentiate into diverse neural cell lineages. During 3 stages of differentiation, CHIR99021, together with SMAD inhibitors, was the key factors for quick induction of ESCs to differentiate into neural rosette-like colonies, which could facilitate neurosphere-forming and promote cell proliferation of NESCs at the second and third stages of differentiation. The generated NESCs were a robust population to be subjected to repeated freeze-thawing and cryopreservation and full differentiation into highly expandable OPCs under the induction of SHH, PDGF-AA and bFGF. The NESCs-derived OPCs could further differentiate into OLs under the induction of T3, cAMP, IGF-1 and NT3. And during the whole differentiation from ESCs into OLs, the detailed gene expression changes confirmed the control by distinct signaling pathways, which might provide an insight into the effects of key growth factors and cytokines on neural regeneration and remyelination in CNS.

Stem cell-based therapy offers great promise for treating traumatic injury and neurodegenerative disorders, which affect neuronal and glial cells in midbrain, hindbrain and spinal cord. The recent iPSC technology induces the generation of patient-specific iPSC-derived cells, which recapitulate a disease model at the molecular level for understanding the mechanisms of CNS diseases in petri-dish [34]. To better use iPSC technology, precise and reliable differentiation strategies are required to avoid unnecessary factors. The current differentiation strategies for human pluripotent stem cells do not lead to a precise and scalable production of pure committed NSCs and OPCs in a relatively short period of time, while mouse pluripotent stem cells used in this study underwent the developmental transitions, thus producing a pure population of expandable mNESCs and OPCs rapidly. The future translation of our finding to humans will contribute to the prospective use of cell-based therapies in the clinic.

## Supporting Information

S1 Fig Phase contrast images showing cell morphology changes during ESCs differentiation by using three inducers Dorsomorphin, SB431542, and noggin. **A**. ESCs on feeder-free ES medium **B**. Neural rosette by adding three inducers Dorsomorphin, SB431542, and noggin in neural basal medium **C**. Neurosphere at the presence of bFGF and EGF **D**. No attachment after dissociation with accutase Scale bar, 400 μm.
(TIF)

S2 Fig Phase contrast images showing cell morphology changes during ESCs differentiation by adding four inducers Dorsomorphin, SB431542, noggin and CHIR99021. **A**. ESCs cultured in feeder-free medium **B**. Neural rosette by adding four inducers: Dorsomorphin, SB431542, noggin and CHIR99021 in neural basal medium **C**. Neurosphere at the presence of bFGF and EGF **D**. All dissociated cells from neurosphere attached to the plate after dissociation of accutase Scale bar, 200 μm.
(TIF)

S3 Fig Phase contrast images of differentiation of NESCs to OPCs under defined differentiation conditions. (TIF)

S1 File (DOC)

S1 Table List of RT-PCR primers. (DOC)

S2 Table List of qPCR primers. (DOC)

S3 Table List of antibodies. (DOC)
