# Supplementary figures and images for: Sequential Differentiation of Embryonic Stem Cells into Neural Epithelial-Like Stem Cells and Oligodendrocyte Progenitor Cells
Source: PLoS One. 2016 May 18;11(5):e0155227. doi: 10.1371/journal.pone.0155227 (PMC4871441; doi:10.1371/journal.pone.0155227)

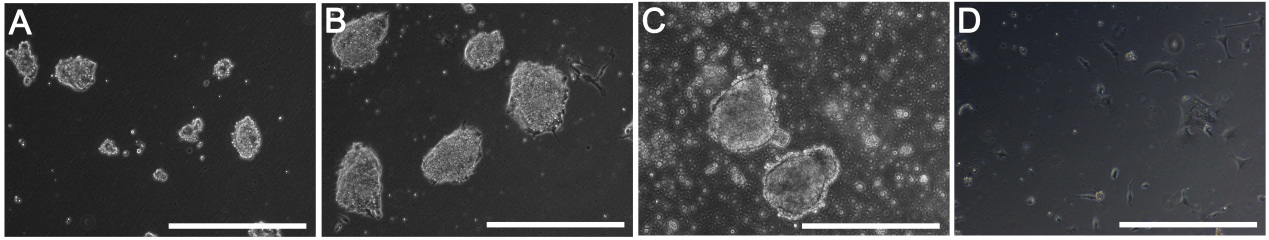

Supplement: S1 Fig — A. ESCs on feeder-free ES medium B. Neural rosette by adding three inducers Dorsomorphin, SB431542, and noggin in neural basal medium C. Neurosphere at the presence of bFGF and EGF D. No attachment after dissociation with accutase Scale bar, 400 μm. (TIF) [file pone.0155227.s001.tif]

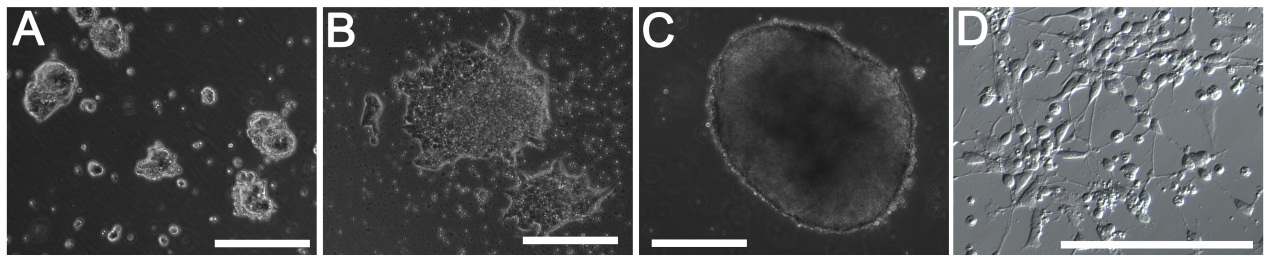

Supplement: S2 Fig — A. ESCs cultured in feeder-free medium B. Neural rosette by adding four inducers: Dorsomorphin, SB431542, noggin and CHIR99021 in neural basal medium C. Neurosphere at the presence of bFGF and EGF D. All dissociated cells from neurosphere attached to the plate after dissociation of accutase Scale bar, 200 μm. (TIF) [file pone.0155227.s002.tif]

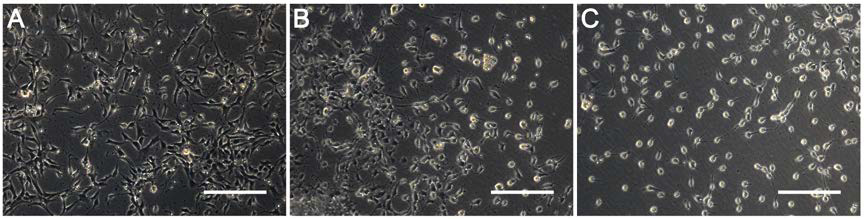

Supplement: S3 Fig — (TIF) [file pone.0155227.s003.tif]
