# Supplementary material for: Sequential Differentiation of Embryonic Stem Cells into Neural Epithelial-Like Stem Cells and Oligodendrocyte Progenitor Cells
Source: PLoS One. 2016 May 18;11(5):e0155227. doi: 10.1371/journal.pone.0155227 (PMC4871441; doi:10.1371/journal.pone.0155227)
Supplement: S1 File — (DOC) [file pone.0155227.s004.doc]

#### Materials and Methods

*Cell Culture of mESCs*

The cells were cultured on irradiated CF1 MEFs with normal mESC growth media, which consisted of knockout DMEM (Gibco) supplemented with 20% KSR (Gibco), 0.1 mM 2-ME (Gibco), 2 mM L-glutamine (Gibco), 0.1 mM non-essential amino acids (Gibco), and 10^3^ units ml^-1^ LIF (ESGRO, Millipore, Bedford, MA), as described previously . For feeder-free culture, cells were grown on gelatin-coated culture dishes in chemically defined media, which consisted of knockout DMEM supplemented with 1×N2 (Gibco), 1×B27 (Gibco), 0.1 mM 2-ME (Gibco), 2 mM L-glutamine (Gibco), 0.1 mM nonessential amino acids (Gibco), 50 μg ml^-1^ BSA fraction V (Gibco), 10^3^ units ml^-1^ LIF (ESGRO, Millipore) and 10 ng ml^-1^ BMP4 (R&D System).

*Isolation and culture of mNSCs*

The pregnant ICR mice were provided by the Experimental Animal Center of Nantong University. The mNSCs were isolated from E13.5 ICR mice from pregnant ICR mice. The pregnant ICR mice were deeply anesthetized with an intraperitoneal injection of xylazine (10 mg/kg), ketamine (95 mg/kg) and acepromazine (0.7 mg/kg) as described previously. Under deep anesthetization, mice were sacrificed. The head of embryo was removed quickly and immerged into phosphate buffered saline (PBS, pH 7.2). Dissected cortical or striatal germinal zone (GZ) tissues were transferred to serum-free media and mechanically dissociated into a cell suspension with a fire-polished Pasteur pipette. After wash with PBS and centrifugation, the cells were seeded on a petri dish in a NSC medium (DMEM /F12, Gibco, supplemented with 1× B27 from Gibco, 20 ng ml^-1^ EGF from R&D Systems, and 20 ng ml^-1^ FGF2 from R&D Systems).

*RNA Isolation, RT-PCR and Quantitative Real Time RT-PCR (qPCR)*

Total RNA was prepared using an RNeasy Plus Mini Kit (QIAGEN, Hamburger, Germany) according to the manufacturer’s instructions. cDNA was generated by reverse transcription of total RNA (1 µg) using Transcriptor First Strand cDNA Synthesis Kit (Roche, Indianapolis, IN). RT-PCR was carried out using DreamTaq DNA Polymerase (Thermo Scientific, Carsbad, CA) in BioRad T-100 PCR machine (BioRad, Hercules, CA). GAPDH was used as an internal control, and samples without reverse transcription were used as negative controls. The amplification products were analyzed by agarose gel electrophoresis. And the RT-PCR primers and conditions are listed in S1 Table.

qPCR was performed and analyzed by using the ABI StepOnePlus™ Real-Time PCR Systems (Applied Biosystems, Waltham, MA) with FastStart Universal SYBR Green Master Rox (Roche) for relative quantification of the indicated genes. The transcript of GAPDH was used for internal reference gene. During the differentiation of mouse ESCs into OLs, the gene expression at different stages during differentiation was compared to the previous stages using ^△△^Ct method for calculation. And the control group (the previous stage) was defined as “zero” to compare the difference (as shown in **Fig 1** and **Fig 2**); While in comparison of NESCs and NSCs, the control group was defined as “1” to be compared with experimental group in ratio as shown in **Fig 4**. The qPCR primers are listed in **S2 Table**. The data were derived from 2 independent experiments, and analyzed by mean ± SEM.

*Immunocytochemical Staining*

Cells were fixed with 4% paraformaldehyde for 15 min at room temperature and rinsed three times with PBS. Spheres were fixed for 45 min, rinsed three times with PBS. Cells were blocked for 1 hr at room temperature with 5% goat serum/0.2% Triton X-100/PBS and incubated overnight at 4 °C with primary antibodies (see **S3 Table** for a complete list) in 2% goat serum/PBS (Triton was omitted throughout for MBP staining). After three washings in PBS, Alexa Fluor secondary antibodies conjugated with either 488, 555, or 647 (ThermoFisher) were applied at dilution ratio 1:1,000 in PBS and nuclei were counterstained with Hoechst 33342 (Sigma) at dilution ratio 1:4,000 for 1 hr at room temperature. Cells were viewed under Microscope (Carl Zeiss GmbH Axio Vert.A1, Jena, Germany) with appropriate filters for cell identification and count. Confocal images were taken under a scanning laser confocal microscope (TCS-NT-UV; Leica, Bannockburn, IL). The percentage of positive cell population was counted in 4 random fields from 2-4 independent experiments. And all the primary antibodies with appropriate dilutions for immunostaining were listed in **S3 Table**. Data were presented as means ± SEM and analyzed by student t-test.

*Migration assay*

In brief, mESCs-derived NESCs (15,000 cells) were plated on insert chamber coated with Matrigel with 8 µm diameter pores (BD Biosciences) in 100µl of control media (DMEM/F12 media,). The bottom wells were added with 600µl of migration media: in DMEM/F12 plus 1×B27 and 1×N2, supplemented with SAG, bFGF, and PDGF-AA or without supplements as control. After culture for 72 hours, the upper chambers were removed and washed in PBS. Cells attached to the upper face of the membranes were gently removed using a cotton scrub. Membranes were then fixed in methanol, processed for Crystal Violet staining and mounted on glass slides. Cells were counted under the Carl Zeiss microscope (bright field, 100×magnification) in 10 random fields/filter, 2 replicates for each experiment. Migration activity was calculated as the mean number of cells/field and expressed as the fold-increase to basal conditions in control medium. Data are expressed as the mean ± SEM, 2-4 independent experiments.

*Flow Cytometry Analysis*

In brief, the cells were dissociated as single cell suspensions, and for cell surface marker detection such as Nestin, the samples were stained with mouse monoclonal antibody of Nestin (Beyotime, Shanghai, China) on ice for 30 min, followed by incubation with FITC-anti-mouse-IgG (Sigma). And negative controls were NESCs stained only with 2ºantibody. For detection of the intracellular antigen, the cells were fixed in 2% PFA and permeabilized by 0.2% Triton-100, and blocked with 5% FBS and then incubated with primary antibodies such as mouse-anti-PLZF followed by incubation of FITC-anti-mouse-IgG (Sigma) as secondary antibody. Cells were then analyzed and quantified by flow cytometer (FACStar Plus Flow Cytometer, BD Biosciences).

**References：**

1. Zhou H, Wu S, Joo JY, Zhu S, Han DW, Lin T, et al. (2009) Generation of induced pluripotent stem cells using recombinant proteins. Cell Stem Cell 4: 381-384.

2. Cheng Q, Jiang C, Wang C, Yu S, Zhang Q, Gu X, et al. (2014) The Achyranthes bidentata polypeptide k fraction enhances neuronal growth in vitro and promotes peripheral nerve regeneration after crush injury in vivo. Neural Regen Res 9: 2142-2150.
