# Supplementary material for: Sequential Differentiation of Embryonic Stem Cells into Neural Epithelial-Like Stem Cells and Oligodendrocyte Progenitor Cells
Source: PLoS One. 2016 May 18;11(5):e0155227. doi: 10.1371/journal.pone.0155227 (PMC4871441; doi:10.1371/journal.pone.0155227)
Supplement: S1 Table — (DOC) [file pone.0155227.s005.doc]

**S1** Table RT-PCR primers

| **Gene** | **Sequence（5′-3′）** | **NCBI ID** | **Length** |
| --- | --- | --- | --- |
| Cdh2 | AGGAACACTGCAAATCTATTTACTT | NM_007664.4 | 481 |
|  | CCGTTTCATCCATACCACAA |  |  |
| Pou3f2 | TTCTGTTTTGTTTTGCCTTATCC | NM_008899.2 | 490 |
|  | CACTGTGTGTTTCCAAGTTCTCA |  |  |
| Ntn1 | TGACACACAGTGGCACAAAGGTA | NM_008744.2 | 594 |
|  | AGCAGAATCGGTGACACAGGAG |  |  |
| Ncam1 | AAGAATCCTGGCATTTCAAGTGG | XM_011242415.1 | 458 |
|  | GGCTGAGGTCCTGAACACAAAG |  |  |
| Ascl1 | GGGAACGAGAGAGCAATTAGAAA | NM_008553.4 | 396 |
|  | AAAAGGAGAGGGGAGAAACGAG |  |  |
| Crabp2 | AATGGGAGAGTGGAAACAAAAT | NM_007759.2 | 450 |
|  | AATCACACAGACTACAAGGATAAGG |  |  |
| Pax6 | CCTGGGACAACACACACTAGATATA | XM_005064824.1 | 438 |
|  | GTGAACAACTGCAAAACACTTAGGT |  |  |
| Rarb | GAGGAACCAACAAAAGTAGACAAGC | NM_001289762.1 | 432 |
|  | CAAAAACTAAGCAGCAGTAAAAATG |  |  |
| Neurod4 | GAAGCTGTGGACAGAGATAGTAGAG | XM_006513142.2 | 433 |
|  | AGAAGAGGAAGAAGATGGAGATAAA |  |  |
| Nestin | AGAGGAAGAAGAAGAGCAGGTGATG | NM_016701.3 | 460 |
|  | TCCCACAGTCTCCAGTGATTCTATG |  |  |
| Nkx2-2 | CATGTCGCTGACCAACACAAAG | XM_006498900.1 | 430 |
|  | CAGCTCGTAGGTCTGCGCTTT |  |  |
| Sox10 | CAGGAAGGGTTAGGGTAGGAGG | XM_006520672.1 | 401 |
|  | GGCTGACAGAAGGCGAGAAGA |  |  |
| Cspg4 | CTGTCTGTTGACGGAGTGTTGAA | NM_139001.2 | 465 |
|  | GCTGTTGCGGAGTAGCATGGTAC |  |  |
| Egfr | TGAAGAAGTGCCCCCGAAA | XM_006514491.1 | 477 |
|  | GCAGCCCCAGTGATGTGATGT |  |  |
| Calcrl | CTTTTCCCACTCTGATGCTCTCC | XM_006499918.1 | 433 |
|  | GTTGTTTGTGCTTATTTTCTTTCCA |  |  |
| Olig2 | CCCTCCTGTTGTCTCTCCTGTTG | NM_016967.2 | 404 |
|  | ATCGCGCATTTCGTTGATATTATAT |  |  |
| Grm5 | TGATGTCGCAGAGGCAGAGGA | XM_006507183.1 | 535 |
|  | GGAGACTGGAGAGTTTGGGGTG |  |  |
| Neto1 | TCAATCCTCTCCCTACCACCGA | XM_006526486.1 | 530 |
|  | CTGAAAATCCCATAGACTCCAGCTC |  |  |
| Pdgfra | GTGCTGTTGGTGATTGTCATTGTCT | XM_006504263.1 | 512 |
|  | TTCTTCGGCTTCTCTGGGTGTT |  |  |
| Olig1 | ACAGGCTCCCTTGCTCTCTCC | NM_016968.4 | 454 |
|  | CTCGCCCAACTCCGCTTACTT |  |  |
| Myrf | GAGGGCAGAGCAAGACCAAGAA | XM_006526934.1 | 551 |
|  | ACACAGGCGGTAGAAGTGGAAGTAG |  |  |
|  |  |  |  |
| **Gene** | **Sequence（5′-3′）** | **NCBI ID** | **Length** |
| Tubb4a | CAGCAAGATCCGAGAGGAGTTTC | NM_009451.3 | 492 |
|  | ACAGCAGCCACAGTCAGGTAGC |  |  |
| Elovl7 | AACTCAAGAAAGCGATGATAACGTA | NM_029001.5 | 471 |
|  | TAGTGACAAGAACAAACTGGACAAG |  |  |
| Mbp | TCCCTTTTCGTTTGAAGATTGAGTT | XM_006526458.1 | 538 |
|  | AGATGGTGACATTTGGCGGC |  |  |
| Mobp | CCCAGTGAGATGAGTCAGAAAATG | NM_001039365.2 | 550 |
|  | TAGAGGGGACAAAAGGCAAGAGAT |  |  |
| Cldn11 | GTGGATTGGGAAAGAAATAAAAGAT | NM_008770.3 | 501 |
|  | ACAGAATGGAGACAGAAATAGAAGG |  |  |
| Mog | TGTTTGTTATTGTGCCTGTTCTTG | NM_010814.2 | 550 |
|  | TATTGTGGGCTCTTCTGTTCCTGA |  |  |
| Enpp6 | GGTGTTGCTCCTGGATGGTTTT | NM_177304.3 | 496 |
|  | GATGCGCTCATGGTATATGGCT |  |  |
| Plp1 | CTCCAACCTTCTGTCCATCTGC | NM_001290561.1 | 445 |
|  | AGCTTTCACTCCTCTGCGACTTG |  |  |
| Mag | CATGGCGTCTGGTATTTCAATAGTC | XM_006539595.1 | 453 |
|  | AGGCACGAAGTGTAGCAGCGAC |  |  |
| GAPDH | GGTGAAGGTCGGTGTGAACGGA | XM_011241212.1 | 369 |
|  | GCAGAAGGGGCGGAGATGATG |  |  |
