# Supplementary material for: Sequential Differentiation of Embryonic Stem Cells into Neural Epithelial-Like Stem Cells and Oligodendrocyte Progenitor Cells
Source: PLoS One. 2016 May 18;11(5):e0155227. doi: 10.1371/journal.pone.0155227 (PMC4871441; doi:10.1371/journal.pone.0155227)
Supplement: S2 Table — (DOC) [file pone.0155227.s006.doc]

**S2** Table qPCR primers

| **Gene** | **Sequence（5′-3′）** | **NCBI ID** | **Length** |
| --- | --- | --- | --- |
| Cdh2 | CCCAAGTCCAACATTTCCATCC | XM_006525553.1 | 190 |
|  | CTTTATCCCGCCGTTTCATCC |  |  |
| Pax3 | TCCGATATTGACTCTGAACCTGATT | XM_011238676.1 | 135 |
|  | CCTGGTGTAAATGTCTGGGTAGTG |  |  |
| Pou3f2 | ATCCACATCAGGAAAGAGGGAATAA | NM_008899.2 | 171 |
|  | AACAAAACAGAACCCAAAGGACAGT |  |  |
| Ntn1 | CCAGGAGAGCCAGTATGGTTTGT | NM_008744.2 | 179 |
|  | GCGTGGGGGAAGTCTAGTTAAGA |  |  |
| Ncam1 | TACCCAAGTGCCACAATCTCCTG | XM_011242416.1 | 172 |
|  | CCTGTCCAATACGGTTCACTGCT |  |  |
| Neurog1 | GACGCCCTGTTTCATCCCATAC | NM_010896.2 | 103 |
|  | CTTCAGCCAGTTCCCCATCTATT |  |  |
| Ascl1 | TCCTGCATCTTTAGTGTTTCTCGC | NM_008553.4 | 103 |
|  | GGTTGGCTGTCTGGTTTGTTTGT |  |  |
| Crabp2 | CTTGCTGCCACTATGCCTAACTTTT | NM_007759.2 | 161 |
|  | GTGTCATTCTCCTGTTTGATCTCGA |  |  |
| Phox2b | TGTGTCTGAGTGACGGGTGTATGT | NM_008888.3 | 146 |
|  | TTCCTTTTGTATCTTTGCTGCTTT |  |  |
| Pax6 | ACAGATTATTATCCGAGGGGGTC | XM_006498915.2 | 146 |
|  | CTGCCCGTTCAACATCCTTAGTT |  |  |
| Rarb | AAAACGACGACCCAGCAAGC | NM_001289762.1 | 151 |
|  | CAGCATTTCCTGAATGAGAGGTG |  |  |
| Neurod4 | CTCTTATGGAATGCTCGGAACCTTA | XM_006513142.2 | 116 |
|  | TCATCTTCTTTTTCTTGGGACCTCT |  |  |
| Nestin | GGAGGAAAGTGTGAAGGCAAAGATA | NM_016701.3 | 128 |
|  | TGCATTCCAGAGTCTCACTGCTAGT |  |  |
| **Gene** | **Sequence（5′-3′）** | **NCBI ID** | **Length** |
| Nkx2-2 | ATCGCTACAAGATGAAACGTGCC | NM_001077632.1 | 184 |
|  | CCCTCAAATCCACAGATGACCAG |  |  |
| Sox10 | AGCAGGCTGGACACTAAACC | XM_006520672.1 | 147 |
|  | AAAGGGGCAGCGATGTGTTA |  |  |
| Cspg4 | GCATCATCATTCCGGTGTGC | NM_139001.2 | 113 |
|  | GGTCAACACCTGGACATCGT |  |  |
| Egfr | CGGACTAGCCATTCTTCCTATGTAT | NM_007912.4 | 140 |
|  | GACAAAGTAGCCCTTCACACCAT |  |  |
