# Supplementary material for: Sequential Differentiation of Embryonic Stem Cells into Neural Epithelial-Like Stem Cells and Oligodendrocyte Progenitor Cells
Source: PLoS One. 2016 May 18;11(5):e0155227. doi: 10.1371/journal.pone.0155227 (PMC4871441; doi:10.1371/journal.pone.0155227)
Supplement: S3 Table — (DOC) [file pone.0155227.s007.doc]

**S3 Table List of primary antibodies**

| **Primary antibody** | **Host** | **Supplier (Cat. No.)** | **Application** |
| --- | --- | --- | --- |
| Oct3/4 | mouse | Santa Cruz Biotech (sc-5279) | 1:50 |
| SSEA-1 | mouse | CST | 1:50 |
| Nestin | mouse | Beyotime (AN203) | 1:10 |
| Sox1 | rabbit | R&D (AF3369) | 1:10 |
| Sox2 | rabbit | ProteinTech (11880-1-AP) | 1:200 |
| Pax6 | goat | Abcam (ab5790) | 1:20 |
| Sox9 | rabbit | Millipore (ab5535) | 1:500 |
| CD133 | rabbit | Bioss (bs-0395R) | 1:100 |
| PLZF | mouse | Santa Cruz Biotech (sc-22839) | 1:50 |
| Dach1 | rabbit | Santa Cruz Biotech (sc-98563) | 1:50 |
| ZO-1 | rabbit | Santa Cruz Biotech (sc-10804) | 1:50 |
| TH | mouse | Santa Cruz Biotech (sc-374047) | 1:50 |
| NeuN | mouse | Millipore (MAB377) | 1:100 |
| 5HT | mouse | Novus(NB120-16007) | 1:50 |
| β-Tubulin III | rabbit | Sigma (T2200) | 1:200 |
| Olig-2 | rabbit | Millipore (AB9610) | 1:50 |
| Sox10 | rabbit | Abcam (ab155279) | 1:250 |
| MBP | mouse | Covance (SMI-99P) | 1:200 |
| GFAP | mouse | BD(556328) | 1:20 |
